# Supplementary material for: Bayesian refinement of protein structures and ensembles against SAXS data using molecular dynamics
Source: PLoS Comput Biol. 2017 Oct 18;13(10):e1005800. doi: 10.1371/journal.pcbi.1005800 (PMC5662244; doi:10.1371/journal.pcbi.1005800)
Supplement: S1 Archive — (BZ2) [file pcbi.1005800.s011.tar.bz2 › gromacs_4.62_bayesian_saxs_refi/share/html/online/g_disre.html]

g\_disre


| g\_disre Main Table of Contents |

**VERSION 4.6  
Sat 19 Jan 2013**

---

### Description

g\_disre computes violations of distance restraints.
If necessary, all protons can be added to a protein molecule
using the g\_protonate program.

The program always
computes the instantaneous violations rather than time-averaged,
because this analysis is done from a trajectory file afterwards
it does not make sense to use time averaging. However,
the time averaged values per restraint are given in the log file.

An index file may be used to select specific restraints for
printing.

When the optional -q flag is given a .pdb file coloured by the
amount of average violations.

When the -c option is given, an index file will be read
containing the frames in your trajectory corresponding to the clusters
(defined in another manner) that you want to analyze. For these clusters
the program will compute average violations using the third power
averaging algorithm and print them in the log file.

### Files

| option | filename | type | description |
| --- | --- | --- | --- |
| **-s** | topol.tpr | Input | Run input file: tpr tpb tpa |
| **-f** | traj.xtc | Input | Trajectory: xtc trr trj gro g96 pdb cpt |
| **-ds** | drsum.xvg | Output | xvgr/xmgr file |
| **-da** | draver.xvg | Output | xvgr/xmgr file |
| **-dn** | drnum.xvg | Output | xvgr/xmgr file |
| **-dm** | drmax.xvg | Output | xvgr/xmgr file |
| **-dr** | restr.xvg | Output | xvgr/xmgr file |
| **-l** | disres.log | Output | Log file |
| **-n** | viol.ndx | Input, Opt. | Index file |
| **-q** | viol.pdb | Output, Opt. | Protein data bank file |
| **-c** | clust.ndx | Input, Opt. | Index file |
| **-x** | matrix.xpm | Output, Opt. | X PixMap compatible matrix file |

### Other options

| option | type | default | description |
| --- | --- | --- | --- |
| **-[no]h** | bool | no | Print help info and quit || **-[no]version** | bool | no | Print version info and quit || **-nice** | int | 19 | Set the nicelevel || **-b** | time | 0 | First frame (ps) to read from trajectory || **-e** | time | 0 | Last frame (ps) to read from trajectory || **-dt** | time | 0 | Only use frame when t MOD dt = first time (ps) || **-[no]w** | bool | no | View output .xvg, .xpm, .eps and .pdb files || **-xvg** | enum | xmgrace | xvg plot formatting: xmgrace, xmgr or none || **-ntop** | int | 0 | Number of large violations that are stored in the log file every step || **-maxdr** | real | 0 | Maximum distance violation in matrix output. If less than or equal to 0 the maximum will be determined by the data. || **-nlevels** | int | 20 | Number of levels in the matrix output || **-[no]third** | bool | yes | Use inverse third power averaging or linear for matrix output |

---

http://www.gromacs.org  
gromacs@gromacs.org
